# Anti-Osteoporotic Effects of Polysaccharides Isolated from Persimmon Leaves via Osteoclastogenesis Inhibition

**DOI:** 10.3390/nu10070901

**Published:** 2018-07-13

**Authors:** Youn-Hwan Hwang, Hyunil Ha, Rajeong Kim, Chang-Won Cho, Young-Ran Song, Hee-Do Hong, Taesoo Kim

**Affiliations:** 1 Herbal Medicine Research Division, Korea Institute of Oriental Medicine, Daejeon 34054, Korea; hyhhwang@kiom.re.kr (Y.-H.H.); hyunil74@kiom.re.kr (H.H.); younme1112@nate.com (R.K.); 2 Korea Food Research Institute, Wanju-gun, Jeollabuk-do 55365, Korea; cwcho@kfri.re.kr (C.-W.C.); Song.Young-ran@kfri.re.kr (Y.-R.S.)

**Keywords:** polysaccharides, *Diospyros kaki*, osteoporosis, ovariectomy, osteoclast differentiation

## Abstract

Persimmon (*Diospyros kaki* L.f.) leaves have traditionally been used as a phytomedicine, in health beverages to treat cardiovascular and respiratory disease and to promote maternal health in East Asia. In particular, polysaccharides from persimmon are known to have anti-coagulant, anti-oxidant, and immune-stimulatory activities. However, their beneficial effects against osteoporosis have not been reported. In the present study, we investigated the anti-osteoporotic effects of polysaccharides from persimmon leaves (PLE0) using an in vivo model of ovariectomy (OVX)-induced bone loss and an in vitro system of receptor activator of nuclear factor-κB ligand (RANKL)-induced osteoclast differentiation. In the OVX mouse model, PLE0 remarkably improved OVX-induced trabecular bone loss by suppressing osteoclast activity. In primary bone marrow-derived macrophages (BMMs), PLE0 dose-dependently inhibited osteoclast differentiation. In addition, PLE0 down-regulated RANKL-induced activation of mitogen-activated protein kinases (MAPKs) such as p38, ERK, and JNK resulting in suppression of nuclear factor of activated T cells cytoplasmic 1 (NFATc1) expression. Our results indicate that PLE0 has anti-osteoporotic effects in OVX-induced bone loss via inhibition of osteoclast differentiation. Taken together, PLE0 from persimmon may prevent postmenopausal bone loss and osteoporotic bone fragility.

## 1. Introduction

Bone remodeling is tightly regulated via coupling and communication between osteoblasts (bone-forming cells) and osteoclasts (bone-resorbing cells). Disruption of bone remodeling is usually due to an increase in osteoclast resorbing activity relative to osteoblast-induced bone formation or due to a decrease in bone turnover where both formation and resorption are reduced, leading to osteoporotic bone diseases [1]. Osteoclasts are multinucleated giant cells formed via proliferation, differentiation, and fusion of mononuclear hematopoietic progenitor cells. During these processes, macrophage colony-stimulating factor (M-CSF) and receptor activator of nuclear factor-κB ligand (RANKL) have pivotal roles in osteoclast formation and activation [2,3]. RANKL, as a key regulator of osteoclast differentiation, triggers the activation of the mitogen-activated protein kinases (MAPKs) and the canonical nuclear factor-κB (NF-κB) pathway through binding to its receptor, RANK. These processes finally up-regulate expression of NFATc1, a master transcription factor of RANKL-induced osteoclast differentiation [4]. Previous reports demonstrated that ectopic expression of NFATc1 causes precursors to undergo efficient osteoclastogenesis without RANKL, and that NFATc1-deficient embryonic stem cells fail osteoclast differentiation by RANKL [5,6]. Given this background, inhibitors of osteoclast differentiation and function can provide a successful strategy to treat bone diseases, and several studies have focused on developing anti-osteoporotic agents, including nutraceuticals, which act via modulation of the above signaling pathways. 

Over the last several decades, there has been increasing evidence showing beneficial effects of herbal beverages and their-derived products, such as Noni leaf, Herba *Epimedii*, *Salvia miltiorrhiza,* icariin and tanshinones in preventing bone loss, and reducing osteoporosis risk [7,8,9,10]. Persimmon (*Diospyros kaki* L.f.) leaves, widely distributed and cultivated in Asia, have been traditionally utilized as an herbal medicine and in health functional beverages to treat cough, internal hemorrhage, hypertension, cerebral arteriosclerosis, and to promote maternal health [11]. Recently, tea and beverages prepared using persimmon leaves have become increasingly popular as a natural food additive due to their health benefits including antioxidant, anti-cancer, anti-aging, and tobacco smoke protection effects, and other medicinal uses [12,13,14]. Previous studies have demonstrated a variety of bioactive components in persimmon leaves that correspond to a broad range of pharmacologic and nutraceutical properties, including flavonoids (kaempferol, quercetin, and their glycosides), terpenoids (oleanolic acid, ursolic acid, and pomolic acid), vitamin derivatives, chlorophyll, kryptoxanthin, and polysaccharides [11]. Polysaccharides from persimmon are a group of hetero-polysaccharides (molecular weight, 1.3 × 10^5^ Da) consisting of arabinose, galactose, glucose, mannose, and rhamnose [15]. Water-soluble sulfated polysaccharides from persimmon have anti-coagulant and anti-oxidant activities [16,17]. Recently, pectic polysaccharides from persimmon leaves were found to have immune-stimulatory effects in RAW264.7 cells, which are a myeloid cell line that can differentiate into osteoclasts. [18,19]. However, effects of persimmon polysaccharides on osteoporosis have not been reported. We investigated the effect of polysaccharides from persimmon leaves (PLE0) in ameliorating in vivo osteoporotic bone loss and inhibiting in vitro osteoclast differentiation.

## 2. Materials and Methods

### 2.1. Reagents and Chemicals

Fetal bovine serum (FBS), α-modified minimal essential medium (α-MEM), bicinchoninic acid assay (BCA) kit, and chemiluminescence reagents were purchased from Thermo Fisher Scientific Inc. (Rockford, IL, USA). Recombinant human M-CSF was kindly provided by Dr. Yongwon Choi (University of Pennsylvania School of Medicine, Philadelphia, PA, USA). Recombinant soluble human RANKL was prepared as described previously [20]. RIPA lysis buffer, and protease and phosphatase inhibitors were obtained from Millipore (Billerica, MA, USA) and Roche Applied Science (Indianapolis, IL, USA), respectively. Cell Counting Kit (CCK)-8 was purchased from Dojindo Molecular Technologies Inc. (Tokyo, Japan). RNeasy kit was obtained from Qiagen (Hilden, Germany). High-Capacity cDNA Reverse Transcription Kit and Taqman probes were purchased from Applied Biosystems (ABI, Waltham, MA, USA). Specific antibodies against phospho-p38 (Thr180/Tyr182), p38, phospho-ERK1/2 (Thr202/ Tyr204), ERK, phospho-JNK1/2 (Thr183/Tyr185), JNK, phospho-p65 (Ser536), p65, were obtained from Cell Signaling Technology (Danvers, MA, USA). Primary antibodies for c-Fos, NFATc1, and β-actin, and goat anti-mouse IgG-HRP and goat anti-rabbit IgG-HRP secondary antibodies were purchased from Santa Cruz Biotechnology (Santa Cruz, CA, USA). Enzyme-linked immunosorbent (ELISA) assay kits for Procollagen type 1 N-terminal propeptide (P1NP) and C-terminal cross-linked telopeptides of type I collagen (CTX) were purchased from Immunodiagnostic Systems Ltd. (Boldon, UK).

### 2.2. Preparation of Polysaccharides from Persimmon (PLE0)

PLE0 (PCT Patent Application PCT/KR2012/010601) isolated from persimmon leaves was provided by the Korea Food Research Institute [15]. Briefly, dried persimmon leaves (1 kg) were placed in 20 L of distilled water (adjusted to pH 4.5 with HCL) and then enzyme-assisted extracted using commercial pectinase (enzyme addition, 1% *v/w* raw material) for 3 days at 50 °C. The enzyme–hydrolysate was heated at 90 °C for 20 min to inactivate the enzyme and centrifuged at 6500× *g* for 20 min to remove un-desirable residues. The supernatant was precipitated by the addition of three volumes of 99% cold ethanol to obtain the polysaccharide fraction. The collected precipitate was dissolved in distilled water and then dialyzed using Spectra/Por membrane (6000–8000 Da molecular weight cut-off; Spectrum Laboratories Inc., RanchoDominguez, CA, USA). Finally, the high molecular fraction was lyophilized to obtain a polysaccharide fraction (PLE0) from pectinase-treated persimmon leaves, and the PLE0 was stored in the dark at 4 °C prior to experimental use. According to the method reported previously [18], the composition of PLE0 is mainly neutral sugars including galacturonic acid, galactose, arabinose, glucose, glucoronic acid, xylose, rhamnose, mannose, fucose (Table 1).

### 2.3. Ovariectomized Mouse Model and Estimated Parameters of Bone Loss

The animal studies were approved by the Institutional Animal Care and Use Committee at the Korea Institute of Oriental Medicine. Female ICR mice (12-weeks-old, 28–32 g) were obtained from Orient Bio Inc. (Seoul, Korea), and were housed with free access to food and filtered-tap water ad libitum under standard conditions (22 ± 2 °C room temperature, 55 ± 10% relative humidity, 12 h light/dark cycle). After at least one week of acclimatization, the mice were either sham-operated or surgically ovariectomized (OVX) after bilateral dorsal incision, as described previously with some modifications [21]. The sham-operated mice (*n* = 10) served as a control. On day seven after the OVX surgery, the OVX mice were randomly assigned to three groups (*n* = 10): OVX/filtered water, OVX/PLE0 100 (100 mg/kg/day), and OVX/PLE0 200 (200 mg/kg/day). PLE0 was dissolved in filtered water and administered through oral gavage once daily for seven weeks. At the end of the experiment, the mice were sacrificed after fasting for 5 h under CO_2_ anesthesia. Blood samples were collected from the caudal vena cava, and centrifuged (8000× *g*, 10 min). Serum samples were stored at −80 °C before the measurement of bone turnover markers. Femurs were quickly collected and used for analysis with micro-computed tomography (μ-CT).

μ-CT scanning of the distal femora was performed using the Quantume GX μ-CT imaging system (PerkinElmer, Inc., Waltham, MA, USA). Alteration of trabecular bone architecture was evaluated using DataViewer software version 1.4.3.2 (SkyScan, Kontich, Belgium). For trabecular bone analysis of the distal femur in all treatment groups, the volume of interest started 1 mm from the lower end of the growth plate, and extended for 140 cross-sections (2.7 mm high). Bone morphometric parameters including bone mineral density (BMD), bone volume per tissue volume (BV/TV), trabecular thickness (Tb. Th), and trabecular number (Tb. N) were calculated. μ-CT analysis was performed by an investigator blinded as to the experimental groups. For the measurement of bone turnover markers, serum levels of PINP (bone formation marker) and CTX (bone resorption marker), were estimated using ELISA kits, following the manufacturer’s instructions.

### 2.4. Cell Culture and Osteoclast Formation

Bone marrow cells were isolated from tibiae and femora of ICR mice (male, 6- to 7-weeks-old, Orient Bio Inc.), and osteoclast formation assays using bone marrow-derived macrophages (BMMs) were conducted as described previously [22]. Briefly, BMMs were cultured and maintained in α-MEM containing 10% FBS, antibiotics, and M-CSF (30 ng/mL). To determine the effects of PLE0 on osteoclast differentiation, BMMs (1 × 10^4^ cells/well in a 96-well plate) supplemented with M-CSF (60 ng/mL) and RANKL (100 ng/mL) were cultured for four days after treatment with PLE0 (1.56–50 μg/mL). Phosphate-buffered saline served as a control. Medium and PLE0 were replaced every three days, and osteoclast formation was observed via TRAP staining. Under microscopic observation (magnification, ×100), TRAP-positive multinucleated (≥three nuclei) cells were counted and were considered as osteoclasts. Cytotoxicity in the BMMs was evaluated using Cell Counting Kit-8 assay per the manufacturer’s protocol. 

### 2.5. Immunoblotting

Immunoblotting was performed as described previously [23]. Briefly, whole-cell lysates were prepared in RIPA lysis buffer containing protease and phosphatase inhibitors, and protein content was determined using a BCA kit (Thermo Fisher Scientific Inc., Rockford, IL, USA). Cellular proteins (30 μg) were separated via SDS-PAGE gel electrophoresis, transferred to a polyvinylidene fluoride membrane, and blotted with specific antibodies at a 1:1000 dilution of antibodies as mentioned in the figure legends. Chemiluminescence signals were obtained using a chemiluminescence reagent (Thermo) and visualized using a ChemiDoc imaging system (Bio-Rad Laboratories, Hercules, CA, USA).

### 2.6. Real-Time Quantitative PCR

Real-time quantitative PCR was performed using an ABI 7500 Real-Time PCR System (ABI, Foster City, MA, USA) with TaqMan probes (i.e., c-Fos, Mm00487425_m1; NFATc1, Mm00479445_m1; Atp6v0d2, Mm00656638_m1; cathepsin K, Mm00484036_m1; DC-STAMP, Mm01168058_m1; 18S ribosomal gene, Hs99999901_s1) and a TaqMan Universal Master Mix. All experiments measuring the gene expression levels were repeated in triplicate. Relative quantification was performed using the ^ΔΔ^Ct method, normalized to a reference gene (18S ribosomal RNA), and the levels were represented using arbitrary units as fold changes.

### 2.7. Statistical Analysis

All data are shown as mean ± standard error of the mean (SEM). The osteoclast differentiation experiments were repeated independently in triplicate. Normal distribution with the Shapiro–Wilk test were tested to select an appropriate statistical approach for each analysis. Parametric analysis was performed with a one-way analysis of variance (ANOVA) and Dunnett’s post hoc test using the software Prism version 5.0 (GraphPad Software, San Diego, CA, USA). A value of *p* < 0.05 was considered statistically significant.

## 3. Results and Discussion

Botanical polysaccharides, used as food and pharmaceutic additives, have been widely investigated due to their various biological activities including modulation of innate immunity, and their relatively low toxicity and adverse effects compared with synthetic compounds [24,25,26]. Recently, the potential of persimmon-derived polysaccharides in regulating bone physiology, including their anti-oxidant and immune-modulatory properties, has been demonstrated [17,18]. In this context, we investigated whether supplementation with PLE0 exhibited beneficial effects on bone pathophysiology, and further elucidated the cellular and precise mechanisms of action that could account for the in vivo observations. 

### 3.1. PLE0 Attenuates OVX-Induced Bone Loss in Mice

**Figure 1:**
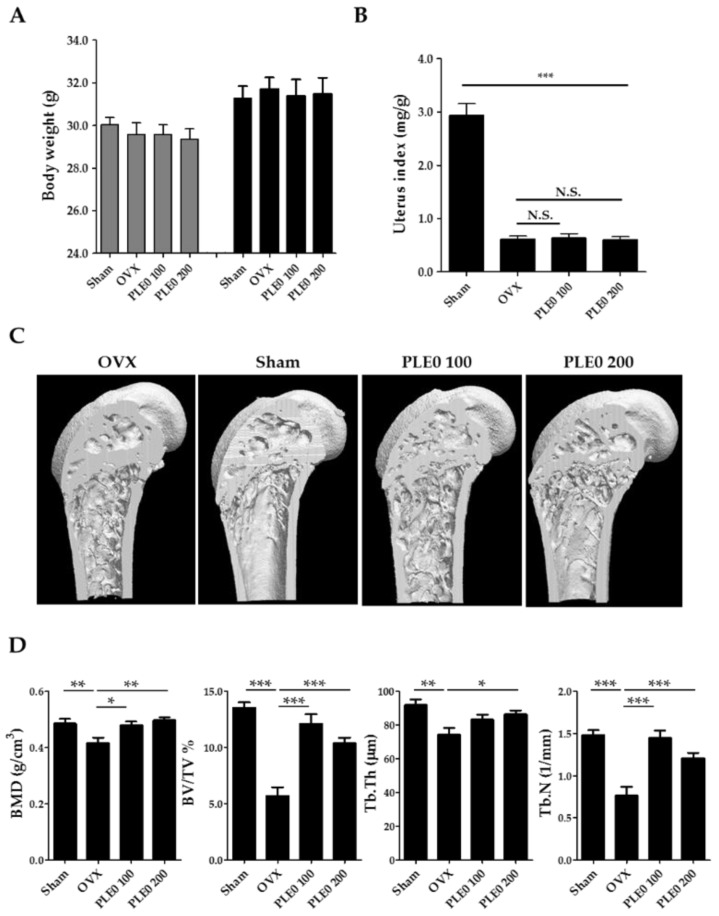
Effects of PLE0 on OVX-induced bone loss in mice (*n* = 10). (**A**) Alteration of body weight, (**B**) relative uterus weight, (**C**) three-dimensional micro-CT images, (**D**) morphometric parameters in micro-CT analysis. The mice were randomly divided into the following four groups; sham-operated/water, OVX/water, OVX/ PLE0 low-dose treatment (100 mg/kg/day, PLE0 100), and OVX/PLE0 high-dose treatment (200 mg/kg/day, PLE0 200). BMD, Bone mineral density; BV/TV, bone volume per tissue volume; Tb. Th, trabecular thickness; Tb. N, trabecular number. All data are represented as mean ± standard error of the mean (SEM), and were analyzed with a one-way analysis of variance (ANOVA) and Dunnett’s post hoc test. *, *p* < 0.05 versus OVX alone; **, *p* < 0.01 versus OVX alone; and ***, *p* < 0.001 versus OVX alone. N.S., no significance.

To determine in vivo anti-osteoporotic effects, we investigated the effects of PLE0 (100 and 200 mg/kg/day) on OVX-induced bone loss in mice, which is a commonly used animal model of postmenopausal osteoporosis. As shown in Figure 1A,B, the body weight of sham-operated, OVX, and/or PLE0-treated mice did not show any significant differences between the beginning and the end of the experiments, but relative uterus weight in the OVX mice was markedly decreased compared to that in the sham-operated mice (*p* < 0.001), indicating uterus atrophy due to OVX-induced estrogen deficiency. 

BMD and morphological observations of bone architecture can provide valuable information in the development of anti-osteoporotic agents [27]. In various osteoporotic animal models regardless of the induction reagents and the causes, morphological alterations are well known to occur in trabecular and cortical bone, and morphometric indices for bone mass and bone formation are decreased concomitantly with the increase in bone resorption [28,29]. In this context, μ-CT-based morphometric analysis may help confirm the efficacy of anti-osteoporotic candidates. Although OVX led to severe impairment of trabecular bones, such as thin and small shape, and plate-like and rod-like trabeculae at the distal femoral metaphysis, oral administration of PLE0 dose-dependently inhibited OVX-induced bone loss (Figure 1C,D). OVX profoundly decreased morphometric parameters including BMD, BV/TV, Tb. Th, and Tb. N in correlation with μ-CT images (Figure 1D). In general, the remarkable reduction of trabecular bone volume was closely associated with a decrease of trabecular thickness and number after OVX [30], indicating damage to the fine network of the numerous trabeculae in the region of the metaphysis via enhancement of osteoclastic bone resorption. PLE0 administration dramatically ameliorated the OVX-related detrimental alterations in the all morphometric parameters as expected, implying protection from bone microarchitecture loss (Figure 1D). 

**Figure 2:**
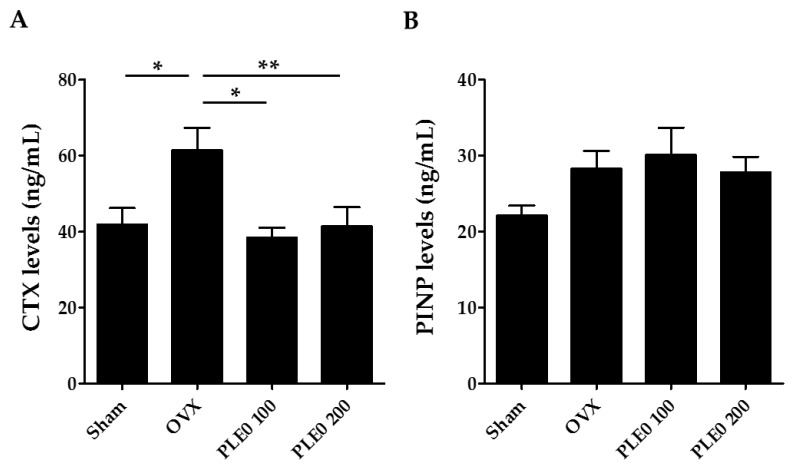
Effect of PLE0 on biochemical bone markers in OVX mice (*n* = 10). (**A**) Serum levels of C-terminal cross-linked telopeptides of type I collagen (CTX); (**B**) serum levels of Procollagen type 1 N-terminal propeptide (PINP). All data are represented as mean ± S.E.M. and were analyzed with a one-way analysis of variance (ANOVA) and Dunnett’s post hoc test. *, *p* < 0.05 versus OVX alone; and **, *p* < 0.01 versus OVX alone.

It has been recommended that serum PINP and CTX be used as reference biochemical markers of bone formation and resorption, respectively [31]. CTX is a type I collagen degradation product whose serum levels are correlated significantly with morphometric measures of bone resorption, whereas P1NP is cleaved from type I pro-collagen during bone formation and the resultant collagen is incorporated into the bone matrix [1,31]. As shown in Figure 2, oral administration of PLE0 markedly modulated excessive CTX levels induced by OVX, but not PINP levels. Thus, our observations suggest that anti-osteoporotic effects of PLE0 are mediated via inhibition of osteoclastic bone resorption rather than affecting osteoblastic function.

### 3.2. PLE0 Inhibits Osteoclast Differentiation in BMMs

**Figure 3:**
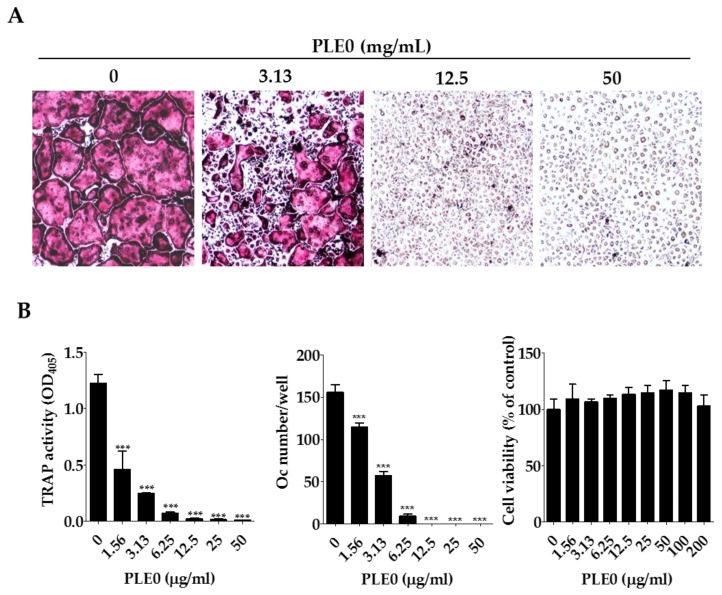
Effects of PLE0 on osteoclast differentiation in vitro. (**A**,**B**) BMMs were cultured with vehicle (distilled water) or PLE0 (1.56–50 μg/mL) in the presence of M-CSF (60 ng/mL) and RANKL (100 ng/mL) for four days. (**A**) Osteoclast differentiation from BMMs in the presence or absence of PLE0 (magnification, ×100); (**B**) TRAP activity, number of cells, and viability of osteoclasts (Oc) after PLE0 treatment (0–200 μg/mL). After fixation and TRAP staining, TRAP-positive multinucleated giant cells (≥ three nuclei) were counted as osteoclasts. All data are represented as mean ± SEM and were analyzed with a one-way analysis of variance (ANOVA) and Dunnett’s post hoc test. ***, *p* < 0.001 versus no PLE0 treatment.

In the presence of two essential cytokines, M-CSF and RANKL, osteoclast precursor cells of the monocyte-macrophage lineage including BMMs differentiate and fuse to form TRAP-positive multinucleated giant cells, which reorganize the actin cytoskeleton to attach to the bone surface and to resorb the bone [32]. To evaluate whether PLE0 affects osteoclast differentiation, BMMs were differentiated with RANKL and M-CSF for four days in the presence of PLE0 (1.56–50 μg/mL), and TRAP activity measurements and osteoclast counting (TRAP-positive multinucleated cells) were performed. As shown in Figure 3, BMMs treated with M-CSF and RANKL showed TRAP-positive multinucleated osteoclast formation. PLE0 dose-dependently inhibited osteoclast differentiation, and the differentiation was completely suppressed at 25 μg/mL PLE0 (Figure 3B). This concentration was used for further mechanistic experiments with PLE0 as described below. To avoid cytotoxicity due to PLE0, cell viability assays were performed under the same conditions. As shown in Figure 3B, PLE0 had no cytotoxic effect even at the highest concentration (200 μg/mL). These findings suggest that PLE0 can directly abrogate RANKL-induced osteoclastogenesis by affecting osteoclast differentiation regardless of cytotoxicity or cell proliferation.

### 3.3. PLE0 Inhibits RANKL-Induced Expression of c-Fos and NFATc1 in Osteoclast Precusor Cells

**Figure 4:**
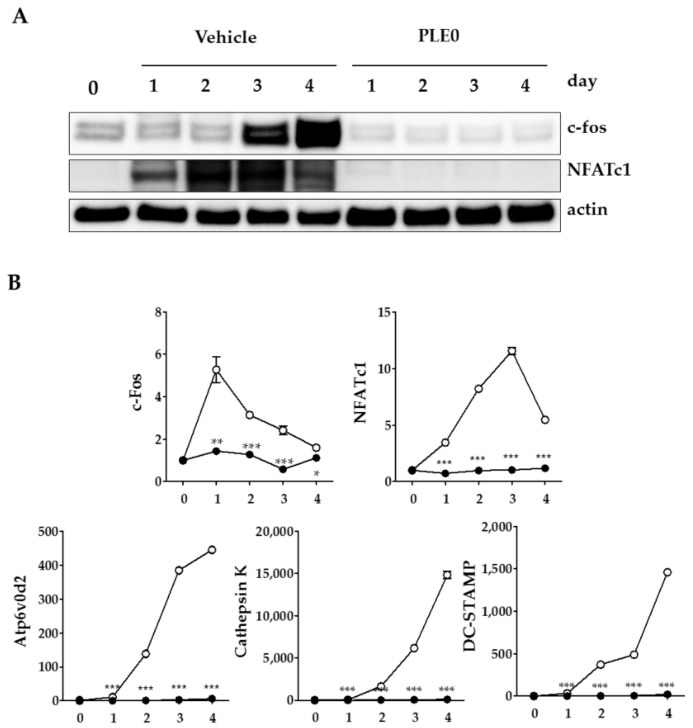
Inhibitory effect PLE0 on RANKL-induced expression of c-Fos and NFATc1 in BMMs. (**A**,**B**) BMMs were treated with PLE0 (25 mg/mL, -●-) or vehicle (-○-) in the presence of M-CSF (60 ng/mL) and RANKL (100 ng/mL) on the various days of incubation. (**A**) Whole-cell extracts were subjected to Western blot analysis with specific antibodies as indicated; (**B**) Total RNA was isolated at the indicated time points and mRNA expression of *NFATc1*, *c-Fos*, *TRAP*, *ATPv0d2*, *cathepsin K*, and *DC-STAMP* was analyzed using real-time quantitative PCR. PCR data are represented as mean ± SEM and were analyzed with a one-way analysis of variance (ANOVA) test and Dunnett’s post hoc test. *, *p* < 0.05 versus no PLE0 treatment. **, *p* < 0.01 versus no PLE0 treatment. ***, *p* < 0.001 versus no PLE0 treatment.

NFATc1 is a master transcription factor in osteoclastogenesis and several transcription factors have been reported to bind to the NFATc1 promoter during osteoclast differentiation [33]. In addition, NFATc1 auto-amplifies itself [2]. Previous studies have demonstrated that c-Fos is a major component of the transcription factor AP-1, and is an indispensable factor associated with early induction of NFATc1 expression in regulating osteoclast differentiation [2,6]. Because PLE0 inhibited RANKL-induced osteoclast differentiation, we examined whether PLE0 can modulate expression of the above transcription factors in RANKL-stimulated BMMs. In Figure 4, the increase in protein and RNA expression levels of c-Fos and NFATc1 during RANKL-induced osteoclast differentiation was significantly suppressed due to PLE0 treatment (25 μg/mL). Because NFATc1 can regulate osteoclast-specific expression of genes involved in osteoclast fusion and maturation including the vacuolar proton pump subunit Atp6v0d2, cathepsin K, and DC-STAMP [4,5], we subsequently confirmed the expression of these genes. As shown in Figure 4B, PLE0 dramatically inhibited the expression of all genes tested in RANKL-stimulated BMMs, indicating that the inhibition of NFATc1 expression in turn resulted in decreased expression of osteoclast-specific genes that encode proteins related to osteoclast differentiation, fusion, and function. Our results indicate that PLE0 inhibits osteoclast differentiation by suppressing NFATc1 and c-Fos expression and therefore NFATc1-driven marker and function genes. However, further studies are needed to elucidate the detailed mechanisms involved in the inhibition of master transcription factors by PLE0.

### 3.4. PLE0 Inhibits RANKL-Induced Early Signaling Pathways

**Figure 5:**
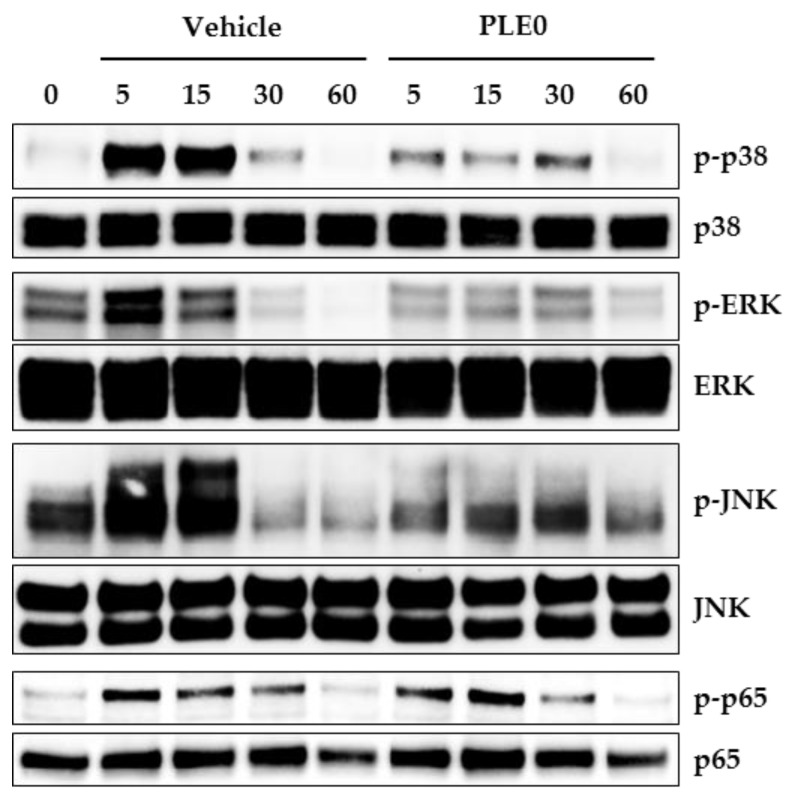
Effects of PLE0 on RANKL-induced activation of MAPKs and NF-kB. RANKL (100 ng/mL)-stimulated BMMs were incubated for the indicated periods of time with or without PLE0 (25 μg/mL). Whole-cell extracts were subjected to Western blot analysis with specific antibodies as indicated. p-JNK, phospho-JNK; p-ERK, phospho-ERK; p-p38, phospho-p38; p-p65, phospho-p-p65.

RANKL binding to its receptor RANK initially activates both the MAPK and NF-κB pathways, which are essential signal pathways in the formation, activation, and survival of osteoclasts [33]. MAPK (p38, ERK, and JNK) activation due to M-CSF and RANKL contributes to the regulation of osteoclast precursor proliferation and osteoclast differentiation by inducing NFATc1 expression [3,34]. Apart from MAPKs, in osteoclast precursor cells, RANKL increases phosphorylation of NF-κB/p65, which translocates to the nucleus and activates transcription of target genes [35]. To gain more mechanistic insights into the PLE0-related inhibition of c-Fos and NFATc1 expression, we determined the effect of PLE0 on the activation of MAPKs and NF-κB/p65. Consistent with previous reports [23,36], RANKL treatment of BMMs resulted in increased phosphorylation of p38, ERK, JNK, and NF-κB/p65 within 15 min after treatment (Figure 5). PLE0 significantly reduced the RANKL-induced phosphorylation of MAPKs (p38, ERK, and JNK), but had no effect on that of NF-κB/p65. Collectively, our results suggest that PLE0 down-regulates c-Fos expression during osteoclast differentiation, at least in part by inhibiting the p38, ERK, and JNK signaling axis.

## 4. Conclusions

We have demonstrated that polysaccharides from persimmon leaves (i.e., PLE0) suppress RANKL-stimulated activation of the MAPK/NFATc1 pathways, inhibiting expression of osteoclast marker genes and finally blocking osteoclast differentiation from BMMs. In vivo, PLE0 also inhibited estrogen deficiency-related bone loss. Therefore, PLE0 may be a reasonable natural alternative to prevent and improve postmenopausal osteoporosis, and further studies addressing its application in bone diseases including rheumatoid arthritis and periodontitis are likely to provide more insight into its anti-osteoclastogenic effects.

## Figures and Tables

**Table 1:** Chemical composition of a polysaccharide fraction (PLE0) isolated from persimmon leaf.

Composition/Component	PLE0 ^1^
Chemical composition (%)	
Neutral sugar	58.1 ± 1.66
Uronic acid	37.0 ± 0.64
2-keto-3-deoxy-mannooctanoic acid (KDO)-like materials	4.43 ± 1.51
Protein	0.48 ± 0.10
Component sugar (mol %) ^2^	
Fucose	2.29 ± 0.50
Rhamnose	4.24 ± 3.80
Arabinose	19.4 ± 4.10
Galactose	26.5 ± 1.42
Glucose	6.77 ± 1.64
Mannose	2.65 ± 0.62
Xylose	4.25 ± 0.19
Galacturonic acid	29.8 ± 1.05
Glucoronic acid	4.33 ± 0.66

^1^ Values are the means of three independent experiments; ^2^ Mol % was calculated from the detected total sugar.
